# Avalanche Photodiodes and Silicon Photomultipliers of Non-Planar Designs

**DOI:** 10.3390/s23125369

**Published:** 2023-06-06

**Authors:** Sergey Vinogradov

**Affiliations:** P. N. Lebedev Physical Institute of the Russian Academy of Sciences, Leninskiy Prospekt 53, 119991 Moscow, Russia; vinogradovsl@lebedev.ru

**Keywords:** APD, SiPM, TAPD, spherical p–n junction, non-uniform electric field, edge breakdown, photon detection efficiency, dynamic range

## Abstract

Conventional designs of an avalanche photodiode (APD) have been based on a planar p–n junction since the 1960s. APD developments have been driven by the necessity to provide a uniform electric field over the active junction area and to prevent edge breakdown by special measures. Most modern silicon photomultipliers (SiPM) are designed as an array of Geiger-mode APD cells based on planar p–n junctions. However, the planar design faces an inherent trade-off between photon detection efficiency and dynamic range due to loss of an active area at the cell edges. Non-planar designs of APDs and SiPMs have also been known since the development of spherical APDs (1968), metal-resistor-semiconductor APDs (1989), and micro-well APDs (2005). The recent development of tip avalanche photodiodes (2020) based on the spherical p–n junction eliminates the trade-off, outperforms the planar SiPMs in the photon detection efficiency, and opens new opportunities for SiPM improvements. Furthermore, the latest developments in APDs based on electric field-line crowding and charge-focusing topology with quasi-spherical p–n junctions (2019–2023) show promising functionality in linear and Geiger operating modes. This paper presents an overview of designs and performances of non-planar APDs and SiPMs.

## 1. Introduction

Certainly, we can track a history of developments of avalanche photodiodes (APDs) and silicon photomultipliers (SiPMs) based on non-planar p–n junctions from the very beginning of the era of semiconductor devices in the 1930s. Many materials (e.g., copper oxide, selenium, diamond, and others) were studied in the photoelectric effects and the feasibility of using them for point-contact and solid-state power rectifiers and diodes.

In the 1940s, silicon and germanium were found to be preferable materials in terms of photoelectric properties and material quality, impurity doping processes for positive (p-type) and negative (n-type) dopants were established, and the silicon photodiode based on a planar p–n junction—“light-sensitive electric device”—was invented by Russel Ohl [1]. In the 1950s, photodiodes of various designs were studied at high reverse voltages, and the main high-field effects were observed, namely, Zener breakdown and avalanche multiplication up to breakdown or microplasma. The breakdown processes often appeared to be facilitated by and localized on some crystal lattice imperfections (e.g., scratches, dislocations, point defects, impurities).

In fact, there was no clear distinction between these processes and their origins, as noted in [2]: “at the time it was not possible to confirm if the avalanche at the location was due to an increased electric field due to nonuniformity of the dopant, carrier tunneling due to traps in the energy bands, band gap narrowing due to lattice distortion, or indeed large crystal misalignments”. Many efforts have been applied to benefit from highly sensitive photodetection with avalanche multiplication in photodiodes (they have become avalanche photodiodes since the invention of the APD by Jun-ichi Nishizawa in 1952 [3]).

To exclude Zener breakdown and defect-related microplasmas as side effects, the developers tried to obtain a pure avalanche multiplication in various ways:

- Making APDs as small as possible for the opportunity to obtain a defect-free active area (e.g., ∼0.03 mm^2^, diameter of ∼0.2 mm) [4,5];
- using a few micrometer light spot for localized studies of microplasmas under controlled photoinjection [5];
- protecting an edge breakdown by mesa structures [4,6] and guard rings [6,7,8].

Providing the most uniform avalanche breakdown in the sensitive area, the planar diode structure with a shallow phosphorus diffusion that forms an avalanche p–n junction in a p-type substrate and with a deep phosphorus diffusion that forms a guard ring became very successful in terms of technology and performance. It was recognized as Ronald Haitz’ APD [5,7] and paved the way for a majority of developments of APDs since the mid-1960s up to modern SPADs and SiPMs.

Another kind of planar APD design, known as a reach-through APD, was developed by Robert McIntyre, Jan Conradi, and Paul Webb [9,10,11]. Instead of the guard ring, the edge breakdown in the reach-through APD is suppressed by partial overlapping of the shallow n+ and p+ layers, forming a high-field avalanche region with uniform avalanche multiplication over this area. The rest of the ultrahigh resistivity silicon substrate of 50–150 μm is fully depleted by a relatively low electric field to improve efficiency (by deeper light absorption) and time response (by faster drift) of the APD in the NIR wavelength range.

Advanced Photonix Inc. introduced an ultimate advancement in the avalanche multiplication uniformity. They achieved the highest statistical uniformity of an n-type dopant spatial distribution due to a neutron transmutation process that converts the silicon isotope to phosphorus and successfully developed large-area APDs (LAAPDs) with areas greater than 1.25 cm^2^ in 1992 [12,13]. Now Advanced Photonix LAAPDs with gains of up to 300 and areas up to 2 cm^2^ are reported as the largest active area devices on the market [14].

Another direction in the development of high-gain APDs in large areas was started in the 1970s as an alternative to p–n junction-based APDs [15]. As noted in [16], “it was first proposed to use a silicon MOS (metal-oxide-semiconductor) structure as an alternative to APDs with a p–n junction. It was advanced that the presence of a silicon dioxide layer with a sufficiently high resistance would effectively stabilize (or limit) the multiplication factor of the avalanche process in the areas of micro-heterogeneities of the semiconductor by accumulation of multiplied charge carriers in such areas.” Indeed, the paper [15] reported on fabrication of large active-area MOS avalanche photodiodes with extremely uniform sensitivity over the entire active area of up to 5 cm^2^, excellent stability, reproducibility, and with a gain greater than 20.

Basic studies of avalanche processes in MOS structures were shortly started at the Lebedev Physical Institute, Moscow, Russia [17,18]. They focused on the negative feedback control over the multiplication process due to accumulation of the avalanche charge at the Si–SiO_2_ interface, which provides a self-stabilized avalanche process with a gain of 10^3^–10^5^. In the mid-1990s these studies advanced to probabilistic consideration of the single-electron avalanche process with negative feedback and resulted in a general concept of the solid state photomultiplier [19,20,21].

MOS APDs were capable of detecting a light pulse only in a short specific time gate under pulsed bias voltage, and this drawback was eliminated in the Metal-Resistor-Semiconductor (MRS) APDs operated in a free-running mode under DC bias voltage [22]. In the first MRS APDs, a resistive SiC layer replaced the blocking SiO_2_ insulator, and the MRS APD was designed as a planar device similar to the MOS APD.

The next generation of MRS APD became a fundamentally non-planar device [23,24,25]. The MRS APD was designed as an array of individual separated p–n junctions formed by n+ diffusion dots of a few micrometers in a p-type substrate to focus the avalanche breakdown at the dots and prevent spreading of the accumulated avalanche over the Si-SiC interface. MRS APDs with very high gain, low excess noise, fast response, and large active area of up to 25 mm^2^ were recognized as promising low-light-level photodetectors, especially since demonstration of their photon number resolution in the late 1990s [26,27]. Despite the good avalanche multiplication parameters, MRS APDs had a low photon detection efficiency (PDE) of about 3%, and this design has been outperformed by planar SiPMs in the 2000s.

The n+ diffusion dots were replaced by separated APD cells based on planar p–n junctions with guard rings. APD cells were connected to a common electrode through individual polysilicon surface resistors and independently operated in a limited Geiger mode. This design appeared to be the most successful in terms of overall performance and reliability, and has been recognized worldwide as SiPM—a new photon-number-resolving photodetector [16,28,29].

The planar p–n junction design became a mainstream in developments of SiPMs as well as conventional linear-mode APDs, GM APDs, SPADs, and SPAD arrays. Today, planar SiPMs are approaching the physical limits of the design performance facing its main inherent trade-off between PDE and the dynamic range [30,31]. This trade-off is especially challenging for NIR SiPM due to the so-called border effect [32].

Despite the planar mainstream, there are known many photodetector designs that have been targeted to utilize some benefits of non-planarity of p–n junctions, non-uniformity of electric fields, small capacitance of sharp-curved electrodes, and other features of non-planar configurations. Some of the designs appeared to be non-competitive, became obsolete, and mostly forgotten (perhaps to be reinvented at the higher technological level or in new application areas). Others demonstrated remarkable advances and became game-changing innovations.

As we see, the planar and non-planar approaches in the APD developments evolve mostly independently but sometimes interfere with and substitute each other in some designs such as planar MOS APDs—non-planar MRS APDs, and planar SiPMs. Today, in electronics and photonics, we try to follow Moore’s law, approaching nanoscale designs where planarity is inevitably destroyed and substituted with overall non-planarity. To anticipate this challenge, this paper presents a review of a variety of non-planar design alternatives. The features, advantages, drawbacks, and finally, competitiveness of different designs are worth considering in a fresh look taking into account recent progress in developments of the non-planar devices, for example, 3D Si sensors and tip avalanche photodiodes (TAPD) with some record characteristics with respect to the relevant planar detectors.

## 2. Non-Planar APD and SiPM Designs

### 2.1. Spherical APD

The so-called “spherical avalanche diode” seems to be the first APD intentionally designed using a non-planar approach [33]. The main idea of the design (Figure 1) was to simultaneously achieve rather contradictory objectives:

- Uniformity of an avalanche multiplication over the sensitive region at the cone tip in the space angle Ω;
- Enhancement of an electric field at the cone tip to lower breakdown voltage;
- Protection from edge breakdown without guard ring structures;
- Reduction of an electric field at the surface of the silicon to lower dark current.

The spherical APD was manufactured with a radius of the p–n junction $r_{0}\approx$ 2 μm and a distance between the cone and the surface d∼ 2–50 μm. An avalanche breakdown at the tip of the cone occurs at a voltage of 50 V (bulk silicon resistivity of 4 Ω·cm) or 85 V (bulk silicon resistivity of 12 Ω·cm). The APD can operate in both a proportional (linear) mode with a gain of ∼200 and a photon counting mode with a gain of ∼10${}^{6}$ by detecting incident photons marked hν with a constant quantum yield within a small sensitive area of ∼50–80 μm${}^{2}$ around the cone with a radius ρ ∼ 4–5 μm. Of course, the design features—backside illumination (BSI), large and deep etched cone, and small sensitive area—appeared to be complicated and impractical to move further with this design.

### 2.2. Metal-Resistor-Semiconductor APD

**Figure 2:**
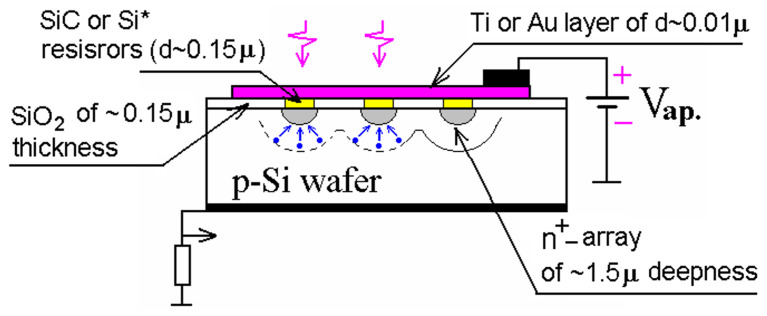
MRS APD design based on n+ diffusion dots with individual SiC or polysilicon (marked as Si${}^{*}$) resistors. Pink arrows represent incident photons, blue dots represent photoelectrons, blue arrows represent drift directions. Reproduced with author’s permission from [44].

The Russian MRS APD evolved from a planar MOS APD first to a planar structure and shortly to a non-planar multichannel or micropixel structure [23,24,34] as shown in Figure 2. Multichannel MRS APD was formed by local inhomogeneities, i.e., n+ diffusion dots of a few micrometer size in a p–Si wafer covered by a thin resistive layer of silicon carbide SiC or polysilicon (marked as Si${}^{*}$). The n+ dots (channels, micropixels) attract electrons by an enhanced electric field at the dot’s curvatures and prevent the spreading of the avalanche charge over the Si–SiC interface by lateral diffusion. An introduction of such artificial local inhomogeneities allows us to eliminate the influence of natural non-uniformity on the avalanche process and stabilize the parameters and reproducibility of the MRS APD. The resistive SiC layer atop the dots provides efficient quenching of the avalanche process in a Geiger mode with negative feedback and complement stabilization of the MRS APD operations against variability of the dot’s breakdown voltages. Low capacitance and low breakdown voltage were identified as the advantages of MRS APD relative to planar APD designs [24,35].

**Figure 3:**
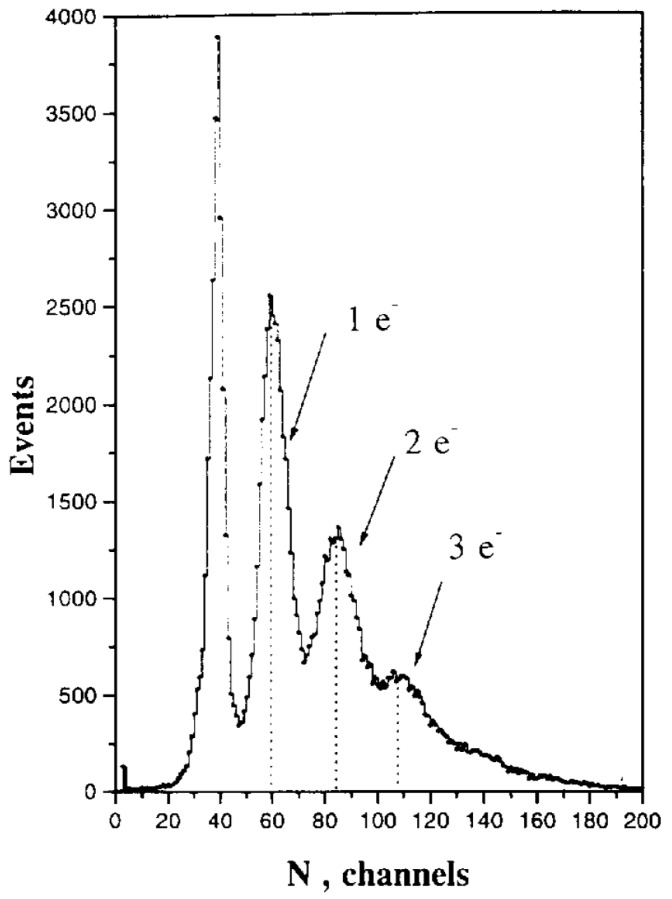
First pulse-height height spectra of a multiphoton LED pulse detection with a photon number resolution by MRS APD at 0 °C. Reproduced with permission from [26]; published by Elsevier, 1997.

Certainly, the most impressive feature of the MRS APD was the unique combination of very high gain $10^{4}$–$10^{6}$ and extremely low dispersion of the gain or excess noise factor due to local negative feedback. This feature breaks the McIntyre law of approximately linear dependence of the avalanche excess noise factor on the mean gain [9,10,36] and results in an unprecedented photon number-resolving detection of multiphoton light pulses. The resolved pulse height histogram (also often called the photoelectron spectrum) of a few detected photons for the first time was observed in [26] at 0 °C (Figure 3) and confirmed at various temperatures including 293 °C in detailed studies [27,35].

However, comparable and even higher performance in photon number resolution and photon detection efficiency than MRS APD was achieved soon by the first planar SiPM developed by the MEPhI/Pulsar team in 2001 [28] utilizing a planar p–n junction design invented by Z. Sadygov in 1996 [16,25].

The non-planar MRS APDs were produced in the 1990s by the Russian Center for Perspective Technologies and Apparatus (CPTA) led by V. Golovin and were considered to be a promising development for some high-energy physics experiments (DESY, CERN CMS). In the early 2000s CPTA developed a new planar design of MRS APD (they also interchangeably call it either SiPM or solid state photomultiplier, SSPM) where for the first time, planar p–n junctions of the pixels on the front surface were separated by deep trenches to suppress an optical crosstalk between pixels as well as to prevent an edge breakdown of the junction [37].

Thus, planar SiPM by MEPhI/Pulsar and CPTA substituted non-planar MRS APD because of higher PDE, lower DCR, lower optical crosstalk, and more reliable production and operation. Since the mid 2000s, planar SiPM has been globally recognized as a new photon-number-resolving avalanche detector of outstanding performance, and the planar SiPM modifications were developed by Hamamatsu [38], SensL [39], ST Microelectronics [40], FBK [41], Excelitas Technologies [42], and KETEK [43] with various combinations of pixel separation by guard rings and trenches.

### 2.3. Micro-Well APD

To outperform both MRS APDs and planar SiPMs in geometric efficiency, therefore, in PDE, Z. Sadygov invented and developed a new original non-planar APD design: the micro-well APD [16,25,44]. Front surface circuitry, quenching resistors, and metal wires for each pixel were completely excluded, providing a geometric efficiency of 100%. The avalanche regions were formed by n+ pixels of a few microns deeply buried in the p-type epitaxial layer (Figure 4). The n+ pixels enhance and focus the electric field like the hemispherical n+ diffusion dots of the MRS APD.

Moreover, the n+ pixels, being micro-wells for electrons in the energy band diagram, collect and trap multiplied electrons while their accumulated charge reduces the electric field and quenches the avalanche breakdown. The small size (low capacitance) and high density of the pixels up to $4\cdot 10^{4}$ mm${}^{-2}$ provide the potential to outperform planar SiPMs in the dynamic range. Indeed, micro-well APD showed record linearity in short-light pulse detection [45].

However, the peak PDE value of ∼35% appeared to be about twice as low as expected at the given quantum efficiency of 80%. Furthermore, micro-well APDs revealed a long pixel recovery time ∼300 μs [45] due to the slow relaxation of the accumulated charge in the micro-wells, eliminating their dynamic range for long light pulse detection.

### 2.4. Discrete Amplification APD

The discrete amplification APD (DAPD) was developed and produced by Amplification Technologies in the 2000s [46,47,48]. DAPDs were based on the concept of solid state photomultiplier (SSPM) as a multichannel APD operated in Geiger mode with single electron negative feedback [19]. DAPD is an array of avalanche amplification channels based on small spatially separated p–n junctions similar to MRS APD as discussed in [49].

In 2009, the DAPD design was implemented with InGaAs/InP heterostructures for the near-infrared (NIR) spectral range [50] and appeared to be the first non-silicon SSPM simultaneously with the negative feedback APD (NFAD) developed by Princeton Lightwave [51].

### 2.5. Avalanche Drift Diode—Back Illumination Drift SiPM

Another approach has been used to achieve an ultimate geometric efficiency of 100% in an attempt to develop the back illumination drift (BID) SiPM. The BID SiPM was designed to combine the concepts of APD and the SDD, utilizing the main benefits of both devices. The SDD is designed to collect electrons from a large depleted volume by lateral drift into a small dot-shaped anode that defines a small capacitance of the SDD [52]. The small capacitance was identified as a primary benefit of the SDD because it allows one to detect low-energy particles with a record threshold sensitivity [53].

Initially, the Max Planck Institute team proposed an SDD-inspired concept of the avalanche drift diode and modeled its structure and electric field distributions [54]. In the next years, they adjusted the implantation profiles to optimize the collection and multiplication of signal electrons in the modified version of the avalanche drift diode, the BID SiPM [55,56], as shown in Figure 5.

The authors presumed that the BID SiPM advantages would be an unstructured thin entrance window with 100% geometric efficiency (fill factor), efficient collection of electrons into a high field region by lateral drift in the focused electric field, and high probability of Geiger breakdown (as only electrons trigger breakdown) and thus, very high PDE overall, as well as short pixel recovery time and low electronic noise due to small diode capacitance.

However, their main concern was an optical crosstalk in this design. Indeed, in the first test structures (made with front entrance windows for a while) the crosstalk effect was estimated to be approximately 54 secondary photons per avalanche event, resulting in a probability of crosstalk of 99.99% by simulation of BID SiPM operations [57]. Faced with such a show-stopper, the team turned from BID SiPM to development of another type of SiPM with bulk integrated quench resistors (SiMPl concept) [58], which is beyond the scope of this paper.

### 2.6. Nano-Multiplication-Region APD

The nano-multiplication-region (later called the nanopillar) avalanche photodiode (NAPD) was invented and developed by the Jet Propulsion Lab, NASA and the UCLA team in 2007 [59,60].

**Figure 6:**
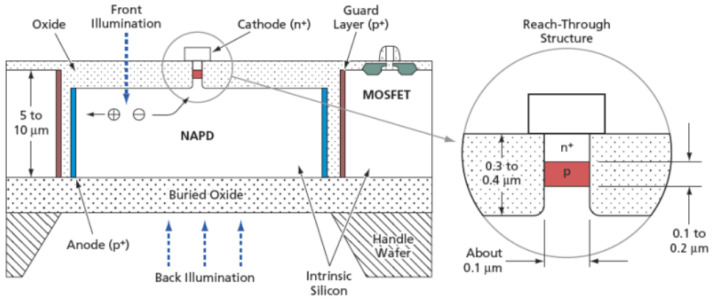
Nano-multiplication-region or nano-pillar APD design. Retrieved from NASA’s Jet Propulsion Laboratory techbrief report at http://www.techbriefs.com/component/content/article/2651 (accessed on 28 May 2023) [62] (public domain).

The design looks like an advance of the ADD or BID SiPM concept toward an ultimately small multiplication region formed by the p–n junction in a nanopillar (Figure 6). Electrons are collected in the nanopillar (in fact, of about 0.1 μm diameter) by lateral drift in the focusing electric field, which is provided by vertical p+ anodes at the pixel borders.

The advantages of the NAPD were expected to be related to the nanoscale size of the multiplication region. The NAPD concept allows to reproduce an approach of so-called separated absorption and multiplication (SAM) APDs (typical for III–V compound materials): a large depleted volume absorbs photons and transfers signal electrons in a relatively low electric field, and the nanopillar p–n junction multiplies the electrons in a high confinement. In fact, a single-electron avalanche process could be localized in a few micrometers or less. As the breakdown is very sensitive to local electric field fluctuations, it is difficult to obtain uniformity of conventional planar-junction APDs and APD arrays, but the nanopillar design eliminates this problem and could be more reliable (e.g., similarly to MRS APD, Section 2.2).

Possible reduction of a filed-enchanced DCR component in the NAPD could also be expected with respect to conventional APDs with a large high-filed avalanche region. On the other hand, we might expect an issue with DCR rather than the improvement. The small size of the high-field avalanche region favors less field-enhanced dark generation, while the large area of the Si–SiO${}_{2}$ interface adjacent to the large sensitive volume with lateral charge transfer favors getting a larger surface contribution to the DCR. Therefore, the overall balance of these contributions could result in a higher DCR per active area compared to the planar APD designs.

The test NAPD samples were made on p-type silicon wafers with resistivity of 80 Ω·cm and with nano-pillar diameter of about 100 nm and height of 800 nm. The measured I–V characteristics could be interpreted as a possible confirmation of “near-constant-gain mode multiplication” predicted by simulations with the measured gain of about 100, but the authors finally noted “However, there remains the possibility that the measured I–V curves do not actually represent the expected gain characteristic.”

This design concept was also applied to 4H–SiC NAPD by some limited simulations using the ATLAS software package from SILVACO (Santa Clara, CA, USA) [61]. No more studies and experimental results have been published on the NAPD to evaluate this original concept in detail.

### 2.7. Germanium APD for Silicon Photonics

Similar emphasis on minimization of an avalanche multiplication region down to sub-micrometer size was revealed in developments of the optical communication receivers in the 2000s. The receiver appeared to be a bottleneck element of an optical data transmission chain in a framework of silicon photonics pursued by IBM, Intel, and some other companies. Contradictory demands of the telecom applications for fastest response and lowest bit error rate eventually necessitate development of the ultra-low-capacitance ultra-short-drift-distance receiver with the fastest internal amplification, namely, the single-carrier multiplication APD. To be compatible with the silicon platform for system-on-chip integration, it was beneficial to make the APD based on a monolithic CMOS-compatible process of Ge/Si technology.

The Intel Silicon Photonics team led by Mario Paniccia developed a conventional separate absorption, charge, and multiplication (SACM) APD structure of a planar design in which light absorption and carrier multiplication occur in a vertical direction inside germanium and silicon, respectively, and achieved a gain-bandwidth product of 340 GHz (maximum measured bandwidth of 11.5 GHz for gains up to 20) for the 30 μm diameter device [63]. The authors assumed that this APD design is feasible for data rates of 40 Gbps.

In contrast, the IBM Watson Research Center team led by Yurii Vlasov developed an original CMOS-compatible lateral receiver design—waveguide-integrated Ge APD as they focused on optical interconnects ranging from data centers and mainframes to board-to-board and on-chip levels ([64], Figure 7). The authors pursued a set of contradictory demands: “ideally the APD should have a compact micrometer-scale footprint, operate at a voltage close to 1 V that is compatible with complementary metal oxide semiconductor (CMOS) technology, possess high avalanche gain, and detect very fast optical signals of up to 40 gigabits per second. These requirements are in strong contradiction to each other and are almost impossible to meet without significant innovations.”

Although Ge APDs suffer from high multiplication noise in germanium making conventional Ge APD noncompetitive for building digital optical links, they invented a new non-planar APD design [65] “to produce a non-uniform electric field in which the high electric field with strength close to avalanche breakdown accelerates electron-hole pairs over impact ionization threshold thus producing avalanche amplification, which is employed as an effective photodetection mechanism or as a current amplification mechanism. The nonuniform field localized with sub-100 nm around the metal contact has field values higher than the impact ionization threshold of the semiconductor material.”

High non-uniformity, high strength, and high confinement of the electric field reduces randomness of the impact ionization process and results in a much lower ENF of the avalanche multiplication than predicted by McIntyre theory for uniform p–n junctions [36] (the corresponding effective ratio of ionization coefficients for electrons and holes $k_{eff}$ is found to be in a range from 0.1 to 0.2 instead of a bulk Ge value of 0.9 as shown in Figure 8).

### 2.8. HgCdTe Electron APD

**Figure 9:**
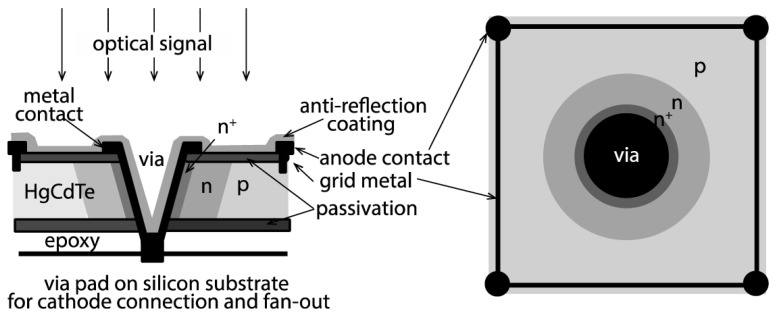
HgCdTe electron APD or high density vertically integrated photodiode (HDVIP) design based on cylindrical p–n junction (side and top views). Reproduced from [70] under open access license (CC BY).

A remarkable example of a record-low ENF in a linear mode APD should also be considered. In 1999, the DRS Technologies team dealing with focal plane arrays with the tunable bandgap semiconductor HgCdTe proposed an APD based on their High density vertically integrated photodiode (HDVIP) [66]. The APD utilized the cylindrical p–n junction architecture as shown in Figure 9. The cylindrical p–n junction favors electron injection from the absorption p-type region into the central high-field n-type region and emphasizes an electron-driven (single-carrier) avalanche multiplication process with low ENF. Hence, this device was called an ‘electron avalanche photodiode’ (EAPD) [66]. Another benefit of the cylindrical junction geometry is a small capacitance which allows us to achieve the bandwidth of 2 GHz (limited by an electron transit time).

However, the main advantage of the EAPD is almost noise-free avalanche multiplication—ENF was observed as low as about 1.3 and independent of gain even at values of about 1000—to be an unprecedented result for linear mode APDs.

This ENF behavior was qualitatively and quantitatively explained by history-dependent ionization theory taking into account a unique conduction and valence band structure of HgCdTe with ballistic electron transport due to low phonon scattering [67,68]. As discussed in [66]: “Ballistic ionization results in a non-random, history dependent, deterministic ionization process, as opposed to the random, history independent process originally treated by McIntyre. The deterministic process thus removes the factor of two in excess noise in the case k = 0 and thus creates the condition of near “noiseless” gain.”

Finally, the cylindrical configuration of the p–n junction has not been found to be an influential factor regarding the unique behavior of the HgCdTe EAPD. Therefore, HgCdTe EAPDs of conventional planar designs have also been successfully developed. Nowadays, planar EAPDs exhibit a lower dark current and technological reliability to be competitive with the cylindrical HDVIP design, having better performance in the SWIR spectral range [69].

### 2.9. 3D Silicon Detector

Solid-state radiation detectors of high-energy particles often have a large thickness of a sensitive region to collect more electron-hole pairs per detected particle. In a planar design of the radiation detector, its electrodes (cathode and anode) are located at the front and back sides of the high-resistivity semiconductor wafer, and the signal charge carriers drift through the whole wafer to corresponding electrodes.

The 3D silicon detector was developed in 1997 with the main objective of turning the drift direction from a long vertical path (backside–frontside) to a short lateral path in a three-dimensional array of electrodes that penetrate into the bulk of the detector while maintaining the same large thickness of the sensitive region in the vertical direction [71].

Non-planar 3D design allows us to reduce the depletion voltages by about two orders of magnitude and the collection distances and times by about one order of magnitude, and greatly increase radiation hardness of the 3D detectors with respect to the planar detectors. Three-dimensional silicon detectors were widely recognized by high-energy physicists and were produced by many technological centers as a valuable instrumentation in many radiation detection experiments (e.g., the double-sided 3D detector for HL-LHC CERN experiments is shown in Figure 10 [72] and reviewed in detail in [73].

An avalanche multiplication in an enhanced electric field near the n+ columns of the 3D silicon detectors has first been assumed [74] and later investigated [75] as a negative side-effect of the 3D design. The avalanche process presumably elongates transit times and complicates charge response calibration. Moreover, as noted in the review [73], “surface breakdown effects prevent from being operated at very large voltages, so that charge multiplication effects can not be fully exploited [76].”

Despite the aforementioned concerns, possible improvement in signal-to-noise ratio (SNR) of the 3D detector response due to charge multiplication appeared to be a rather attractive goal for a dedicated feasibility study. Charge multiplication was especially demanded, as the wafers become thinner to minimize particle scattering, and the thinner the wafer, the lower the absorption and collected charge.

The study on this topic was based on numerical simulations and was reported at the 8th “Trento” workshop on Advanced Silicon Radiation Detectors in 2012 [77]. The authors analyzed various designs to adapt the 3D detectors to operate with built-in charge multiplication along with the entire length of the n+ columns.

First, they faced an early breakdown on the front and back surfaces of the n+ column if the column penetrated the sensitive layer from top to bottom. To suppress the front-surface breakdown, they applied p-spray and field plate measures but did not reproduce the same on the back side expecting technological issues. Therefore, they solved this problem in two steps: (1) lifting the n+ column at about 15 μm to avoid surface breakdown and (2) rounding the sharp edges of the tip of the n+ column and increasing its radius to increase the breakdown voltage at the tip to be comparable to the breakdown along the column.

Regretfully, to the best of the author’s knowledge, no experimental results have been reported on this original design so far. Another contribution on this topic by Marco Povoli et al. “Feasibility Study of Charge Multiplication by Design in Thin Silicon 3D Sensors” was submitted to and IEEE Nuclear Science Symposium in 2019 but it has not been published and its content is not available.

### 2.10. Tip APD

Since early developments of SiPMs based on planar p–n junctions, the planar design provided higher peak PDE ≈15% [28] (2001) compared with the non-planar n+ dot design of MRS APDs ≈10% [35] (2000). In a decade, the maximum PDE of planar SiPMs reached a level of ≈60% [78] (2012) outperforming non-planar micro-well APDs (PDE ≈40% for MAPD-3NK [79], 2018). Therefore, there are no non-planar SiPMs on the market now.

However, since the mid-2010s, improvements in planar SiPM performance have started to approach inherent limits of the planar design. SiPM as an array of APD cells based on planar p–n junctions with an inactive border area reveals several major bottlenecks.

- The first issue is a trade-off between geometric efficiency (and, hence, PDE) and dynamic range: the larger the cell size, the higher ratio of active area to inactive area (PDE), and the lower total number of cells per SiPM area, i.e., the dynamic range of the SiPM. Moreover, larger cells with larger capacitance have a longer recovery time, affecting the dynamic range in CW and long light pulse detection modes [30,31].
- The second issue is an inclusion of a probability of avalanche triggering into PDE as well as into probability of correlated noise events (crosstalk and afterpulsing): the higher the PDE, the higher the correlated noise up to unacceptable values and the so-called second breakdown effect. Regarding optical crosstalk, this problem is partially eliminated by metal-filled trenches at the cell borders, but this measure emphasizes the first trade-off [30,31].
- The third issue in the development of planar NIR SiPM with an extended spectral range to NIR wavelengths is the so-called border effect: the deeper the sensitive epilayer for more efficient the NIR absorption, the larger the insensitive volume at the edges of the p–n junction with losses of NIR efficiency [32].

Therefore, the demand for further advancements in the development of high-dynamic-range SiPMs with high PDE in a wide spectral range could hardly be met with known SiPM designs.

To overcome the limitations of planar SiPMs and the drawbacks of non-planar SiPMs mentioned above, a spherical junction-based SiPM—tip avalanche photodiode (TAPD), has recently been developed [80,81,82] (Figure 11). The TAPD design approach transforms the edge breakdown problem into a benefit of highly efficient collection and multiplication of photoelectrons in the focusing electric field, features low breakdown voltage and low capacitance, and eliminates the need for borders between the cells. When designing SiPM as an array of small tip-like p–n junctions, we can select a pitch size about equal to a depletion diameter to get a low or negligible electric field at a half-pitch distance between tips. In general, it allows us to eliminate the need for a peripheral separation of SiPM cells and their avalanche regions by excluding an inactive dead area between cells and improving a fill factor.

TAPD significantly outperforms modern planar SiPMs. TAPD single electron response (SER) of 4.3 ns fall time is the fastest of the same 15-μm pitch SiPMs due to the lower capacitance of the TAPD (Figure 12a). The peak PDE value of 73% is the highest value among the most efficient large-pitch SiPMs due to higher geometric efficiency (Figure 12b).

Moreover, NIR PDE of 22% at 905 nm is also higher than that of modern NIR SiPMs for LIDAR applications developed by Broadcom (PDE = 18% [84]), On Semiconductor (PDE = 18.5% [85]) and Hamamatsu (PDE = 9% for MPPC S15639–1325PS) due to a 12-μm-thick epilayer without borders between cells.

Recently, the record-high PDE of the TAPD has been confirmed and its relatively good stability in a wide temperature range from −30 °C to 70 °C was reported in [86] (Figure 13a).

Another recent study of TAPD clarified its radiation-induced degradation characteristics [87]. As observed, under irradiation with neutrons up to a fluence of 10${}^{12}$ cm${}^{-2}$, the DCR (including correlated noise) of the TAPD increases from 1 MHz to 2 GHz (estimated) while the DCR of a standard KETEK SiPM of the same 15 μm pitch and the same 1 mm${}^{2}$ area increases from 0.4 to 30 GHz (estimated), i.e., it becomes approximately 15 times higher in an absolute value (Figure 13b).

The radiation hardness of TAPD is assumed to be associated with a strong dependence of the electric field on the junction curvature (almost insensitive to irradiation) and its relatively weaker dependence on the doping concentration of the p-type epilayer (affected by irradiation due to a so-called acceptor removal effect; see, e.g., [88]), while the planar SiPMs are rather sensitive to doping concentration.

### 2.11. Current-Assisted APD and SPAD

Current-assisted APD (CA-APD) was proposed and developed by the Vrije University team in 2019 [89]. The main goal of the design was to resolve a trade-off of conventional planar APDs where the area of avalanche multiplication is as large as a photosensitive detector area; thus, the detector capacitance is high and the detector response is slow.

The authors noted that the CA-APD concept was inspired by the following: “Separate absorption and multiplication (SAM) regions can be introduced, as was presented in III–V semiconductors. Guiding minority carriers using a drift field has also been implemented to improve detection speed. Electron multiplication in HgCdTe APDs with ‘p-around-n’ geometry for midwave infrared detection has also been reported.”

The CA-APD concept was soon been extended to Geiger mode operations to develop Current-Assisted SPAD (CA-SPAD) in 2020 [90] (Figure 14). In fact, the current-assisted design also reminds the silicon drift detector, the avalanche drift diode, namely, a simplified version of the SDD due to an exclusion of deep implantations of n+ and p+ and external guard rings beyond R${}_{1}$ (see Section 2.5, Figure 5).

Despite the aforementioned simplifications, the CA-SPAD design provides an effective collection of photoelectrons from a large photosensitive volume to a small avalanche region due to the properly configured electrostatic potential (conduction band energy) profile as shown in Figure 15 [90]. Most electrons are collected by drifting directly into the potential well at the n+ cathode, and the minority of electrons from the surface region exhibit slow diffusion passing near the potential wall at the p+ anode.

In the latest development, CA-SPAD has been improved by substituting an on-chip quenching resistor with an active quenching circuit [91]. A peak photon detection probability (PDP) of 56% was achieved for a wavelength of 705 nm and PDP ≈20% for a wavelength of 940 nm. However, a single photon time resolution (jitter) of 194 ns for 785 nm is not as good as typical jitter of modern planar SPADs with thin epilayer, and it has a long pronounced tail due to the diffusion.

### 2.12. Dot APD and Multi-Dot APD

**Figure 16:**
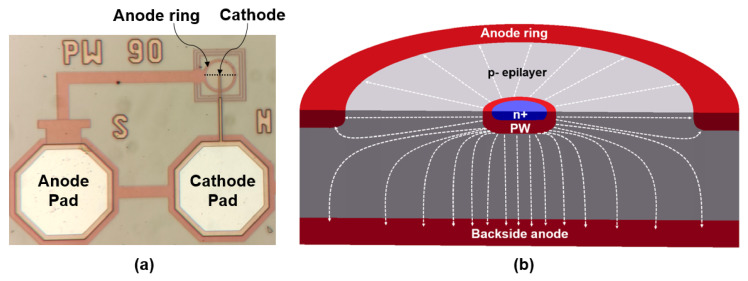
Dot APD design: (**a**) top view on the dot APD; (**b**) cross-section of the dot APD fabricated in 0.35 μm CMOS technology (the cathode radius is 0.9 μm, the total diode radius is 14 μm, and the capacitance is 0.65 fF). Reproduced from [94] under open access license (CC BY).

**Figure 17:**
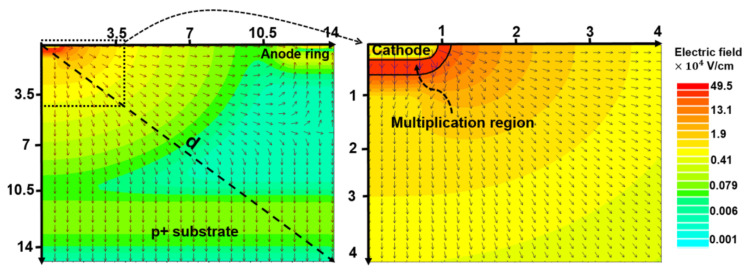
Dot APD electric field-line crowding: 2D electric field distribution across the dot APD shown in Figure 16 (TCAD simulations at 24.5 V). Reproduced from [94] under open access license (CC BY).

**Figure 1:**
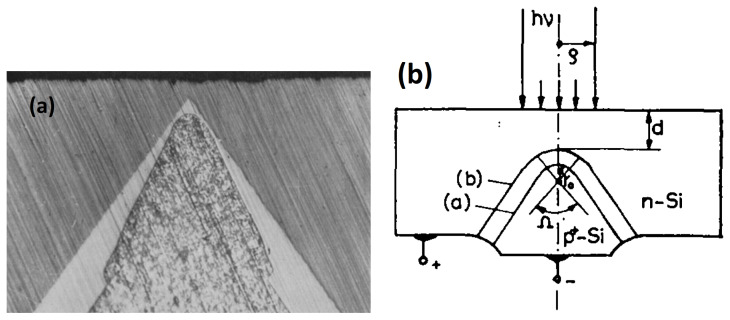
Spherical APD design: (**a**) the n−Si monocrystal substrate, the cone of Au-Si eutectic granular p+Si, and the bright zone of recrystallized p+Si diffusion; (**b**) the APD layout: the metallurgical p–n junction is marked by the line (a) and the depletion region is marked by the line (b); see explanations of other symbols in the text. Reproduced with permission from [33]; published by Elsevier, 1968.

**Figure 4:**
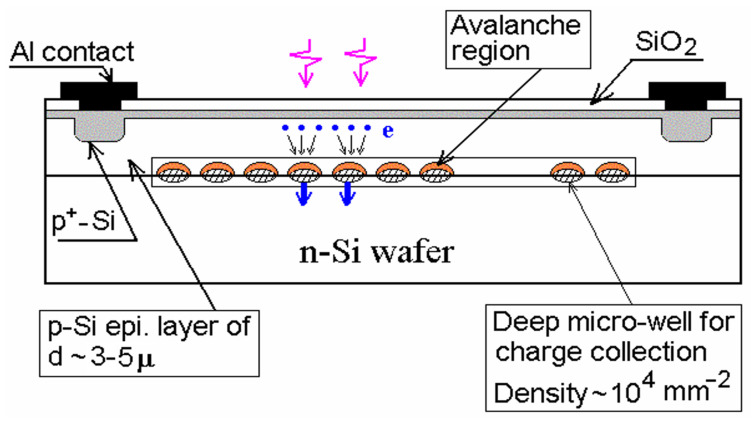
Micro-well APD design based on micro-wells (pixels) of n+ dots buried into p-type epitaxial layer. Pink arrows represent incident photons, blue dots represent photoelectrons, black and blue arrows represent drift directions. Reproduced with author’s permission from [44].

**Figure 5:**
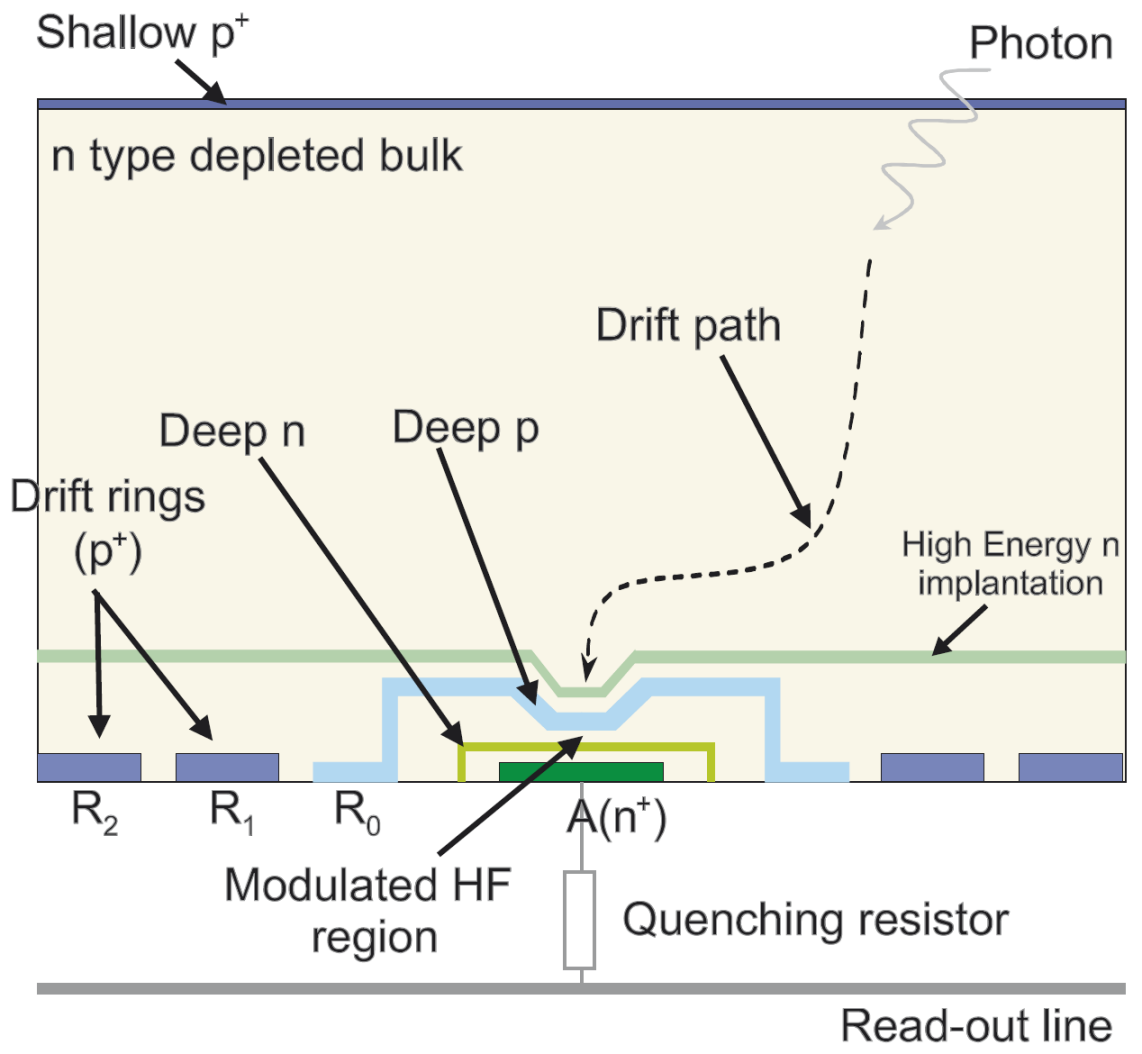
Back illumination drift SiPM inspired by avalanche drift diode design. Reproduced with permission from [56]; published by Elsevier, 2007.

**Figure 7:**
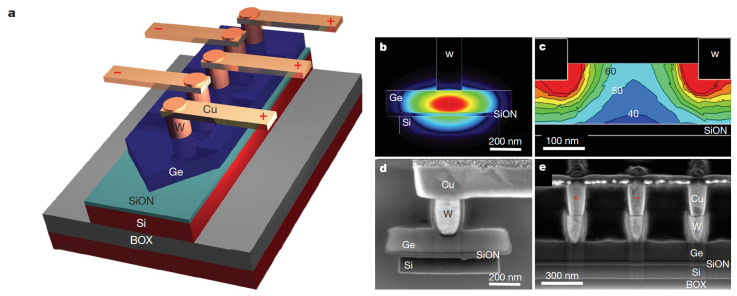
Waveguide-integrated Ge APD design where the absorption and avalanche multiplication Ge layer is deposited on top of a SiON insulating layer which overlays an Si waveguide: (**a**) schematic layout; (**b**) optical intensity profile; (**c**) electric field distribution profile at 2.8 V bias between contacts; (**d**) SEM image of a lateral cross-section of the Ge APD normal to the waveguide; (**e**) SEM image of the longitudinal cross-section of the Ge APD along the waveguide. Reproduced with permission from [64]; published by Springer Nature, 2010.

**Figure 8:**
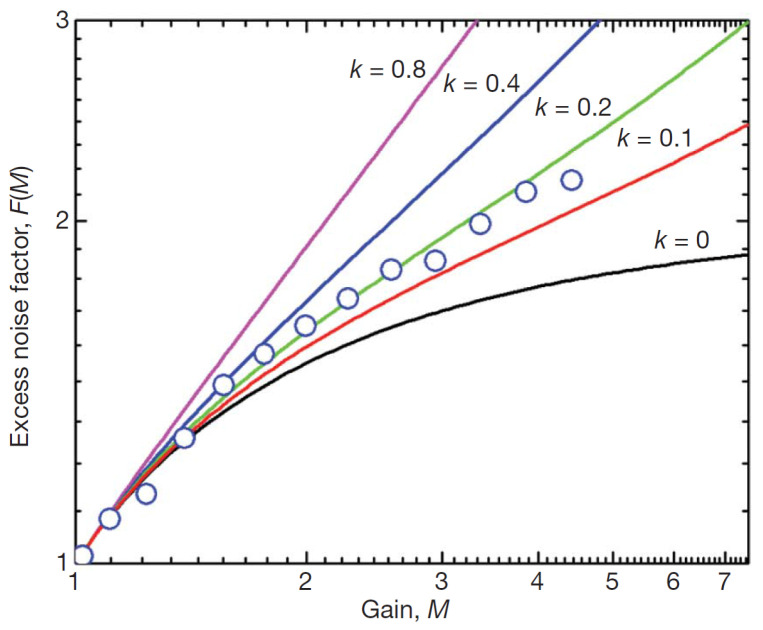
Waveguide -integrated Ge APD (300 nm contact spacing): dependence of the excess noise factor (ENF) on gain (blue circles represent experimental data, colored lines represent calculations by McIntyre theory marked by corresponding $k_{eff}$ values). Reproduced with permission from [64]; published by Springer Nature, 2010.

**Figure 10:**
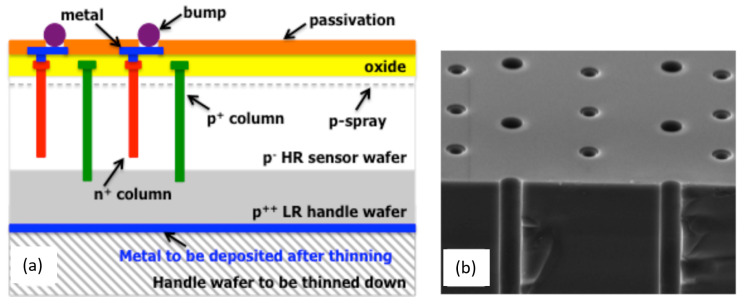
Three-dimensional silicon detector design: (**a**) cross-section of double-sided 3D silicon detector modification for HL-LHC CERN experiments; (**b**) SEM micrograph of two sets of columns etched by deep reactive ion etching. Reproduced with permission from [72]; published by Elsevier, 2016.

**Figure 11:**
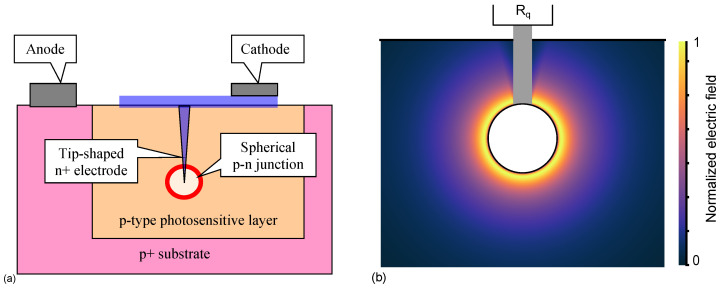
Tip APD design: (**a**) concept of TAPD cell. Reproduced with permission from [83]; published by Elsevier, 2023. (**b**) 2D electric field distribution in the TAPD cell. Reproduced with permission from [81]; published by IEEE, 2020.

**Figure 12:**
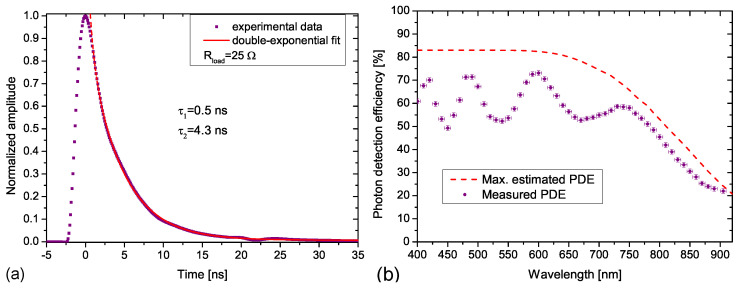
Performance of 15-μm pitch, 1 mm${}^{2}$ Tip APD: (**a**) single electron response temporal profile; (**b**) spectral dependence of measured PDE and maximal estimated PDE taking into account only losses related to geometric efficiency of 83% and light absorption in 12-μm-thick silicon epitaxial layer. Reproduced with permission from [81]; published by IEEE, 2020.

**Figure 13:**
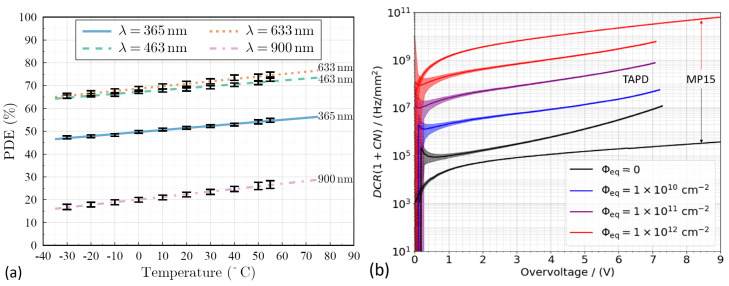
Features of Tip APD: (**a**) dependence of PDE on operating temperature for different wavelengths. Reproduced from [86] under open access license (CC BY). (**b**) Dependence of estimated DCR including correlated noise on overvoltage in comparison with KETEK SiPM of the same 15-μm pitch. Reproduced with permission from [87]; published by IEEE, 2023.

**Figure 14:**
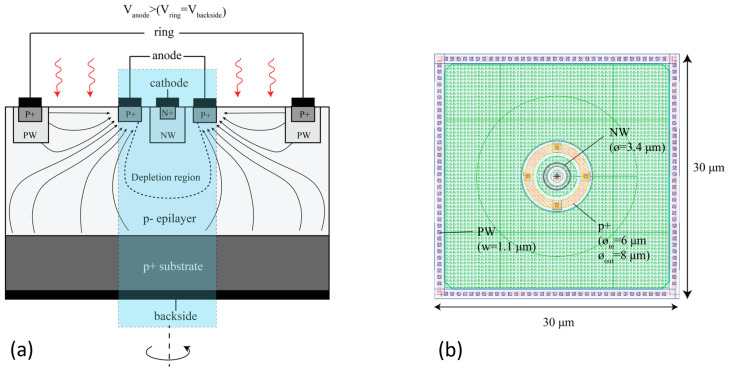
Current-Assisted APD and SPAD design: (**a**) the device cross-section where depletion region boundary is roughly represented by dotted lines and lines with arrows represent the drift field direction for electrons; (**b**) top-view image of the device from layout with illustrations marking the doping layers and dimensions. Reproduced from [90] under open access license (CC BY).

**Figure 15:**
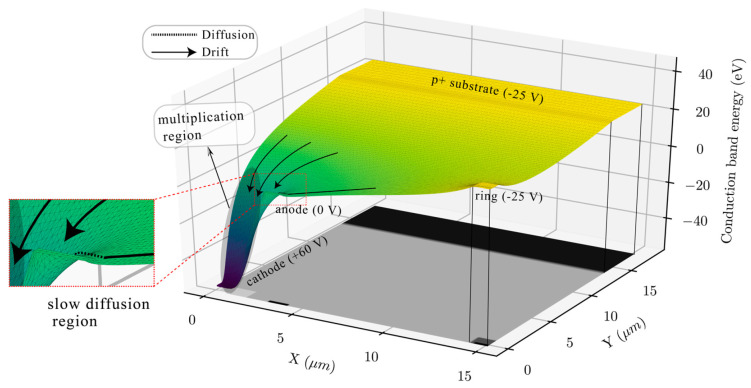
Simulated 3D conduction band energy profile of the Current-Assisted SPAD. Reproduced from [90] under open access license (CC BY).

Referencing current-assisted APD and TAPD, a simplified PIN and APD design based on a small dot-shaped n+ cathode with a quasi-spherical p–n junction (Figure 16) and a focused electric field profile (Figure 17) has been evaluated by a team from Technische Universität Wien led by H. Zimmermann. The authors specified an electric field-line crowding configuration as the main feature of the design.

First, the development of the PIN dot (spot) photodiode was mainly focused on its ultralow capacitance of approximately 1 fF combined with a relatively large sensitive area of 707 μm${}^{2}$ [92,93]. The PIN photodiode has the following dimensions: n+ dot radius of 1 μm, p+ anode inner radius of 15 μm, and epilayer thickness of 24 μm. However, the nine-fold reduced capacitance compared with the planar PIN photodiode does not result in an increased bandwidth (∼300 MHz) due to enlarged transit distances, while rise and fall times are acceptable short of about 1 ns. The photodiodes are expected to be useful as large-area low-noise optical receivers up to 500 MHz.

Second, the dot APD was manufactured using a standard CMOS process without any modifications. It has about the same dimensions as the PIN photodiode presented above: n+ region with a radius of 0.37 μm embedded in an n− well region with a radius of 0.6 μm, p+ anode inner radius of 19 μm, and thickness of the epilayer of 24 μm. The presented APD achieves a gain-bandwidth product of up to 384 GHz, corresponding to the gain of 80 and bandwidth of 1.28 GHz. As summarized in [93]: “The field-crowding APD reduces the optical power and increases the avalanche gain as well as the bandwidth compared to the other APDs.”

**Figure 18:**
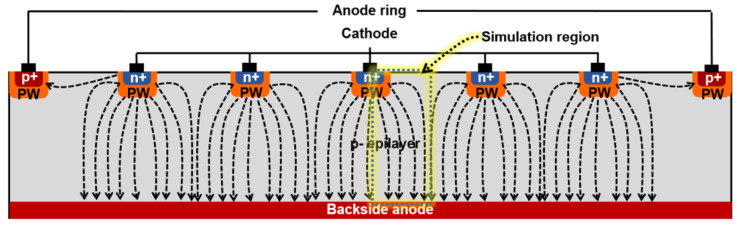
Multi-dot APD design: the array of n+ dot cathodes surrounded by the anode ring. Reproduced from [94] under open access license (CC BY).

Third, the multi-dot APD design as an extension of the same approach has been evaluated by TCAD simulations and manufactured in 0.35 μm CMOS technology as an array of 5 × 5 cathode dots with a pitch of 14 μm (Figure 18). The multi-dot APD is characterized by a gain of 36 and a 3-dB bandwidth of 1.8 GHz while its capacitance is four times lower than that of comparable planar APDs.

### 2.13. Charge-Focusing SPAD

Anticipating a demand for the development of SPAD arrays with a small pitch and high sensitivity to near-infrared (NIR) radiation, especially for LIDAR applications (see, e.g., [95,96]), a team from Katholieke Universiteit Leuven and IMEC (Belgium), and OmniVision Technologies (USA) started R&D on CMOS compatible NIR-enhanced silicon BSI SPAD in 2019 [97].

We have already discussed this problem for the NIR SiPM: the trade-off between high NIR efficiency (thick epilayer) and high dynamic range (small pitch) in the presence of the border effect, which has been resolved in the borderless TAPD design (Section 2.10).

The charge-focusing SPAD design utilizes a small dot-shaped n + cathode with quasi-spherical p–n junction as already discussed in Section 2.12. The authors underlined that the device relies on geometry for the formation of a field peak near the cathode.

It is worth noting that one of the consequences of the geometry-driven electric field configuration appeared to be a low sensitivity of the APD to process fluctuations observed with the first experimental samples [97]: “The variability of the breakdown voltage on a BSI wafer is measured to be less than 0.6%. This is more than an order of magnitude lower than the variability measured for a reference NIR-enhanced SPAD on the same wafer.” This feature allows the development of a reliable and reproducible SPAD array based on the charge-focusing approach.

The first charge-focusing backside-illuminated (BSI) SPAD array prototypes of 3 × 3 and 3 × 10 pixels with active quenching have been developed and measured in 2022 [98].

The BSI SPAD has a peak PDE of 66% near 660 nm and a value of 27% at 905 nm. The results demonstrated that the charge-focusing BSI SPAD outperformed previously reported current-assisted FSI SPAD (PDE of 27% vs. < 11% at 905 nm, DCR of 640 Hz vs. 4 MHz accordingly (Table 1 in [98])). However, the quantum efficiency of the frontside-illuminated (FSI) photodetectors is lower than that of the BSI photodetectors for any comparable designs.

Comparing the charge-focusing SPAD array with the TAPD [81], the authors noted their similar advantages due to the field-line crowding effect and underlined a simple planar technology of the SPAD array preferable for integration with active CMOS electronics.

Let us also note the similarity of the charge-focusing SPAD array and the MRS APD.

### 2.14. Charge-Focusing SPAD Image Sensor

The development of image sensors is a mature and still emerging area of cutting-edge technology and strong competition. To succeed in it, many challenging and often contradictory demands have to be achieved: high PDE in a wide spectral range, low dark and readout noise, high dynamic range starting from single photons, high spatial resolution (micrometer and even submicrometer scale of pixels), high temporal resolution (timing jitter) and large number of pixels in the sensor array [99,100]. Fast progress and remarkable advancements in all these objectives have been demonstrated by a team from AQUA lab, EPFL, and Canon Inc.

First, considering a problem of the miniaturized SPAD array with high fill factor (geometric efficiency), K. Morimoto and E. Charbon proposed a novel guard-ring-sharing technique to resolve the trade-off between fill factor and pixel pitch [99]: “Compared to the conventional well-shared structure, the isolation well between neighboring pixels is eliminated. The pixel is virtually isolated by the shared guard ring region with shallow trench isolation (STI). Assuming the shared guard-ring width of 1 μm, the theoretical limit of the pitch of the pixels can be reduced to 2 μm for 20% fill factor.”

**Figure 19:**
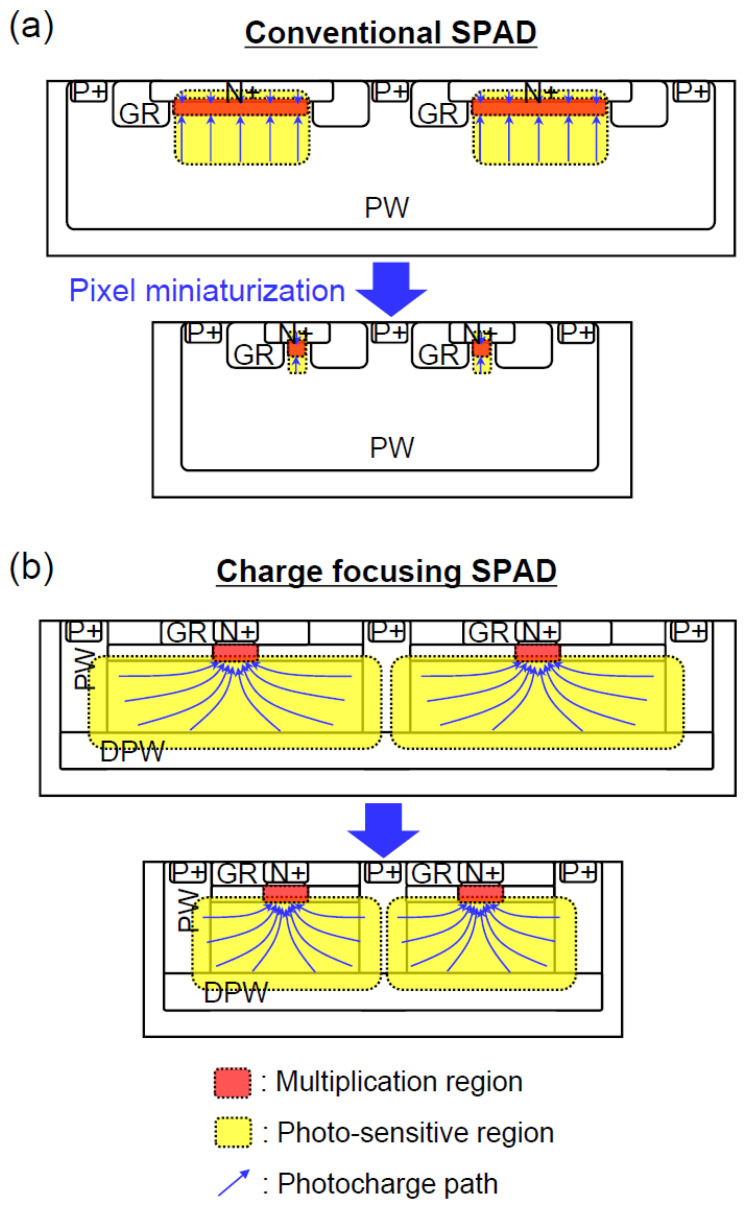
Charge-focusing SPAD concept combining guard-ring sharing and current-assisted/charge-focusing approaches in comparison with a conventional SPAD. (**a**) Schematic cross-section of conventional SPAD arrays with large pixel pitch (top) and small pixel pitch (bottom). (**b**) Schematic cross-section of charge focusing SPAD arrays with large pixel pitch (top) and small pixel pitch (bottom). Red and yellow regions depict multiplication region and photo-sensitive region, respectively. Blue arrows show charge carriers paths. Reproduced from Kazuhiro Morimoto Ph.D. Thesis at https://infoscience.epfl.ch/record/283481 (accessed on 28 May 2023) [102] (public domain).

Second, current-assisted and charge-focusing designs have been identified as another option for miniaturization without loss of a sensitive area. Combining the advantages of guard-ring sharing and charge-focusing approaches, a charge-focusing SPAD image sensor for low-light imaging applications was proposed and developed in 2020 [101,102] (Figure 19).

The following year Canon announced the successful development of a 13.2 mm × 9.9 mm BSI SPAD sensor with the world’s highest resolution of 3.2-megapixel images and high color reproduction even in dark environments [103]. Sensor parameters are the best ever reported for BSI SPAD arrays: highest PDE (peak PDE = 69.4% at 510 nm, NIR PDE = 32.8% at 850 nm and 24.4% at 940 nm), lowest DCR of 44 Kcps/mm${}^{2}$ (comparable with the best planar SiPMs), lowest pixel pitch of 6.39 μm, and lowest timing jitter of 100 ps FWHM.

Recently, this team demonstrated improvements of the SPAD image sensor in dynamic range (143 dB) and power consumption of 0.37 W while relaxing in the pixel numbers (960 × 960 array) and pitch (9.585 μm) [104]. As reported, the fill factor of ∼100%, the PDE of ∼70%, and the low DCR represent the best-in-class performance thanks to the charge-focusing approach. Additionally to charge-focusing BSI configuration, the SPAD pixels are equipped with light-focusing microlenses to generate a majority of photoelectrons near a central axis of the pixel and minimize the transit distance dispersion, i.e., timing jitter.

### 2.15. Nanophotonic SiPM—Quantum Silicon Detector

Initially, a unique photon number resolution of the SiPMs and a very good energy resolution of high energy particles in scintillation detectors with the SiPM readout have been recognized. Since the early 2010s, attention to SiPM applications has been extended beyond energy-resolved to time-resolved or combined ones. Amongst many time-of-flight (TOF) applications, the TOF PET appeared to be the most attractive for many reasons, and TOF-PET/MRI and TOF-PET/CT systems based on SiPMs have been developed by General Electric, Philips, Siemens, and United Imaging.

In these scanners, the TOF modality provides considerable improvements in the image quality by rejecting background events and increasing the signal-to-noise ratio of the image (100 ps TOF resolution results in a 5-fold improvement in SNR or, equally, in a 25-fold dose reduction). However, an ultimate goal in TOP PET is to achieve direct 3D reconstruction of the PET images eliminating the back-projection problem. This goal requires a TOF resolution of 10 ps (equal to a spatial resolution of 1.5 mm along the line of response) [105,106].

To pave the way toward 10 ps TOF-PET as well as other challenging TOF applications like LIDAR and 4D imaging calorimeter for high-energy particle detectors of modern colliders, a team from CERN CMS/Crystal Clear collaboration led by P. Lecoq started various activities on radical improvements of timing performance of SiPMs and scintillation detectors in the mid-2010s, namely, the Brainstorming Workshop on factors influencing the timing resolution of SiPMs, 2015 [107], FAST—Fast Advanced Scintillator Timing EC COST action, 2015–2018 [108], the 10 ps TOF-PET challenge contest [109], and the PHOTOQUANT project of ATTRACT-EU program [110].

**Figure 20:**
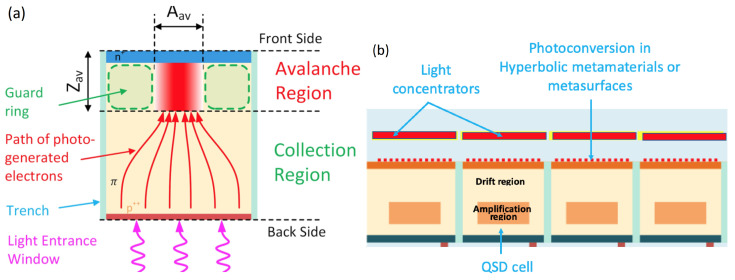
Nanophotonic SiPM—quantum silicon detector: (**a**) concept of the pixel design; (**b**) schematic layout of the array with nanophotonic light concentrators as microlenses and metamaterials as scintillators. Reproduced from [111] under open access license (CC BY).

In a framework of the PHOTOQUANT project, they tried to combine all possible measures to obtain the best TOF resolution from nanophotonic, metamaterial, charge-focusing SPAD and SiPM technologies [111] (Figure 20). The detector concept implements the following ideas:

- Optical focusing of the photons to very small SPADs to fight against the fill factor losses;
- Electron focusing on an even smaller amplification region, to further reduce the capacitance and to reduce the effects of field non-homogeneities;
- Enhancement of the photonic density of states in a well defined depth of the structure (actually in a thin hyperbolic metamaterial layer), to increase the probability of photon-electron coupling (and therefore the PDE through the QE) and to reduce the time jitter by forcing the start of the avalanches at a well defined depth, particularly important for LIDAR applications.

Now, Fondazione Bruno Kessler is investigating the feasibility of this concept for manufacturing using 2.5D and 3D integration technologies, as recently reported [112]. However, they identified several technological challenges to be solved.

## 3. Discussion

### 3.1. Features of Non-Planar Designs

Let us review the features, advantages, and drawbacks of the non-planar designs. As we can conclude from examples of the devices discussed above, their developers paid attention and set priorities, emphasized, or eliminated some of the factors from a limited set of inherent features of non-planar configurations of electrodes, p–n junctions, doping profiles, and electric fields and potentials.

#### 3.1.1. Breakdown Voltage

Low breakdown voltage is an obvious feature of non-planar designs compared to planar designs. For example, the breakdown voltage for a doping concentration of 10${}^{15}$ cm${}^{-3}$ in sharp p–n junctions is about 300 V for planar junctions and about 40 V for spherical junctions with a radius of about 1 μm (these values can be estimated using expressions from the original paper [113] or the handbook [114]). This reduction is especially important for radiation detectors with a thick high-resistive sensitive layer, for example, 3D silicon detectors (Section 2.9).

Moreover, the breakdown voltage has a much weaker dependence on the doping concentration. For example, the breakdown voltage for a doping concentration of 10${}^{14}$ cm${}^{-3}$ is approximately 2000 V for planar junctions and about 50 V for spherical junctions with a radius of approximately 1 μm. As considered in Section 2.13, this feature reduces the breakdown voltage variability over a wafer by about an order of magnitude compared to planar design and improves the reliability and reproducibility of the charge-focused SPAD array.

We can also guess that this effect facilitated the reliable manufacturing of MRS APD in Russia in the 1990s.

In many cases, these features are a strong pure advantage, and we could hardly find some drawback examples.

#### 3.1.2. Electric Field

Enhancement of the electric field at curvatures means a higher field at the same applied voltage with respect to the planar electrodes. The electric field profile has a more pronounced peak at the spherical p–n junction and a steeper decay in the bulk compared to the planar p–n junction. A critical electric field value of the avalanche breakdown (as well as the breakdown voltage) is defined by an ionization integral over a drift path of the charge carriers, i.e., an electric field line; however, it is mostly studied for planar p–n junctions of various 1D profile [115].

Thus, the enhanced electric field reveals itself in three main consequences: (1) low breakdown voltage and (2) the small volume of high-field avalanche region, and (3) large electric field gradients in the avalanche region.

#### 3.1.3. Excess Noise Factor

Excess noise factor F(M) of a stochastic multiplication process with a random gain *M* is an important figure of merit for PMTs and linear mode APDs. Let us note, the APDs outperform the photodiodes only due to low ENF at high gain. ENF is defined by a relative dispersion of the random variable *M*.

(1) $$F\left(M\right)=1+\frac{Var(M)}{{\bar{M}}^{2}}.$$

The ENF features were comprehensively studied since the 1960s starting from R. McIntyre’s basic theory of avalanche multiplication in uniformly multiplying APD [9,36], where the dependence of F(M) on a mean gain $\bar{M}$ for a single carrier (namely, electron) initiated avalanche process was derived as follows:

(2) $$F\left(M\right)=k_{eff}\cdot \bar{M}+\left(2-1/\bar{M}\right)\cdot \left(1-k_{eff}\right),$$

where $k_{eff}$ is an effective ratio of ionization coefficients (holes to electrons).

Main improvements of basic theory were developed to account for a dead-space affected (history-dependent) ionization rate [116,117,118,119] and a mixed-carrier initiation of the avalanche process starting from an arbitrarily specified location of primary carrier within the APD [117,120,121].

In general, if a model includes all relevant effects pointed out above, a good agreement of the simulation results with experiments can be obtained with some fitting of the model parameters (see, e.g., [121]). However, as emphasized by R. McIntyre in his comprehensive study [118]: “A conclusion of this work is that an ionization coefficient is not a fundamental material characteristic at a specific electric field and that any experimental determination of ionization coefficients is valid only for the particular structure on which the measurement was performed.”

Indeed, we can find a variety of values of $k_{eff}$ in experiments with different thickness of avalanche region, conditions of carrier injection, and electric field profiles. For example, in some studies of silicon APD, the values of $k_{eff}$ are reported to be as high as 0.22 [122] and 0.4 [121], while typical values for a pure electron injection are in a range from 0.01 to 0.02.

In contrast with widely studied planar APDs, we can hardly find just a few publications somehow related to the ENF of non-planar APDs [64,66,123,124].

The extremely low ENF of the EAPD (Section 2.8) due to $k_{eff}\approx$ 0 is explained by the band structure of HgCdTe [66]. The key feature of HgCdTe is a very low effective mass of the electrons and a much higher effective mass of the holes resulting in a threshold energy for impact ionization of about a bandgap energy E${}_{g}$ for the electrons and about 2·E${}_{g}$ for the holes.

Low ENF of the waveguide-integrated Ge APD (Section 2.7) which corresponds to $k_{eff}$ < 0.2 while bulk Ge has $k_{eff}$ = 0.9 is attributed by the authors [64] to three effects known for planar APDs:

- dead-space affected ionization rate (emphasized by a nanoscale confinement of the multiplication region);
- hot-electron initiation of the avalanche (electrons have high initial energy entering multiplication region at saturation velocity);
- large electric field gradients in the multiplication region could result in the fast acceleration of secondary carriers towards the ionization threshold as modeled in [125].

Theoretical studies [123,124] focused primarily on the multiplication of avalanches in curved p–n junctions, revealed a considerable reduction in ENF. The ENF reduction is found to be originated from and explained by the dead-space and electric field enhancement effects.

Therefore, there are no observations on some specific effects originated from non-planarity of APD designs solely related to curvature of p–n junctions and divergence of electric fields. We might conclude that the non-planar design with sharp curvatures, enhanced electric fields, and high electric field gradients in a small avalanche region inevitable favors the ENF reduction and makes it more pronounced with respect to the comparable planar design.

#### 3.1.4. Depletion

The depletion width of the p–n junction defines a photosensitive volume with a drift collection of charge carriers. Photon absorption and conversion in this region means drift-limited transit times (much faster than diffusion-limited ones outside of the depletion) resulting in a fast response and low timing jitter of the photodetector. As long as the electric field at the curvatures exhibits steeper decay in the bulk, a radial size of the spherical depletion region is obviously smaller than the depletion width of the planar p–n junction at the same voltage.

However, the difference is not as dramatic as it could be expected. For the previous example of the doping concentration of 10${}^{14}$ cm${}^{-3}$ at a voltage of 50 V, the depletion width of the planar p–n junction is approximately 25 μm while the depletion radius of the spherical one is approximately 10 μm, that is, the diameter of the spherical depletion region is 20 μm.

#### 3.1.5. Dark Noise

The dark generation of charge carriers resulting in dark noise is one of the most uncertain questions in this comparative analysis.

In general, there are contradictory effects with unknown overall balance:

- Pronounced peak of the electric field should increase a field-enhanced dark generation (band-to-band and trap-assisted tunneling, Pool-Frenkel effect);
- The small volume of the high-field avalanche region should decrease the same field-enhanced generation;

In particular, some benefits in dark generation have been expected or observed in the developments discussed in Section 2.1, Section 2.6 and Section 2.8. Remarkably, the most comprehensive of the designs, the charge-focusing SPAD image sensor, demonstrates exceptionally low dark noise (Section 2.14). Some other evaluations are negative.

Therefore, this questionable topic could hardly be clarified for certain in a the near future given the wide variety of designs and technologies of the developments.

#### 3.1.6. Correlated Noise

Avalanche multiplication is associated with specific noise of false correlated events due to crosstalk and afterpulsing. To reduce optical crosstalk, the pixels in modern SiPM are separated by trenches filled with metal or polysilicon to prevent the firing of neighboring pixels by secondary photons (emitted from the primary avalanche location).

Certainly, the borderless version of the non-planar designs is a questionable option for low-crosstalk demanded applications. However, as far as the crosstalk is proportional to the avalanche gain, hence, defined by low pixel capacitance, the relative impact of crosstalk on the performance could be overweighted by high efficiency of the non-planar design.

Afterpulsing is determined by the concentration of deep trapping cites in a vicinity of the avalanche region and its reduction provided by technological improvement. However, as well as the crosstalk, the afterpulsing is proportional to the avalanche gain, and its severity could be not critical due to the low capacitance of the pixel.

#### 3.1.7. Capacitance

The low capacitance of a detector $C_{det}$ and/or its pixel $C_{pix}$ is an obvious feature of the non-planar devices compared with the planar devices. It is considered a very important advantage almost in all studies discussed above, and sometimes appeared to be a primary driving force for device development (e.g., see Section 2.12). The main reason for that is the dependence of timing performance on the capacitance.

For SiPM and SPAD with passive quenching on a resistor $R_{q}$, the capacitance of the pixel directly defines the rise time ≈ $R_{pix}$·$C_{pix}$ where $R_{pix}$ is the internal resistance of the pixel. It is of great importance for TOF applications.

The pixel capacitance also directly defines the pixel recovery time ≈ $R_{q}$·$C_{pix}$—a critical parameter for high dynamic range applications. The slow component of the response fall time is equal to the pixel recovery time.

The detector capacitance defines the fast component of the response fall time ≈ $R_{in}$·$C_{det}$ where $R_{in}$ is the input resistance of an acquisition circuit. This component typically limits a response bandwidth, causing a pile-up effect.

The detector capacitance also contributes to an equivalent noise charge proportional to a sum $C_{det}+C_{in}$ where $C_{in}$ is the input capacitance of the acquisition circuit. However, typically it has a weak influence on the signal-to-noise performance of APDs and, especially, SPAD and SiPM devices as a result of their high gain.

The gain of the SiPM and SPAD devices with passive quenching is proportional to $C_{pix}$, therefore, if the gain appears to be below 10${}^{5}$, it could be an issue that makes acquisition more noisy and complicated.

#### 3.1.8. Efficiency

First and foremost, with respect to the FSI technology typical for the modern SiPMs, the non-planar designs without dead area at the pixel borders can benefit from much higher geometric efficiency compared to the planar devices at the same pixel sizes and pitches. For example, the geometric efficiency of borderless TAPD is 83% and the peak PDE is 73% [81], while the best planar SiPM NUV-HD series from FBK with the same pitch of 15 μm has a geometric efficiency of about 55% and a peak PDE of 35% [126]. KETEK SiPM PM3315-WB series has a bit lower geometric efficiency and peak PDE of about 30%.

The geometric efficiency (fill factor) of any FSI photodetector is finally limited by a dead area of bias/readout wiring network on its front surface.

However, a new trend in the developments of SiPMs and SPAD arrays, the BSI technology, provides an ultimate geometric efficiency of about 100%, thus eliminating such advantages of non-planar designs. Nevertheless, the very promising BSI-based developments discussed above utilize non-planarity for other advantages of this approach.

#### 3.1.9. Dynamic Range

The number of pixels in a multipixel detector is a main parameter which defines a dynamic range, i.e., the number of detected photons causing saturation of the detector response. In case of a short light pulse detection (pulse width less than recovery time), the saturation level is about equal to the number of pixels $N_{pix}$. The non-planar designs without dead area at the pixel borders can benefit from smaller pixel sizes and pitches, and higher pixel densities at the same efficiency with respect to the planar architectures.

In the case of a long pulse and continuous light detection (pulse width longer than recovery time), the dynamic range of the intensity of the detected photon flux at the saturation level is defined by the total intensity of pixel recovering ≈ $N_{pix}$/($R_{q}$·$C_{pix}$).

In a photon-counting mode, the response fall time (mostly fast component) defines a maximum count rate of photons limited by a pile-up effect.

#### 3.1.10. Timing

Timing performance in general is combined by contributions from charge carrier transit processes in the sensitive layer and transient charging-recharging processes in an electric circuit elements of the detector and front-end electronics.

If the photosensitive volume is fully depleted, the collection charge transit times of planar and non-planar devices are defined by the configuration of the volume and should not be different for the same dimensions. In case of partial depletion, the charge collection includes diffusion process which results in a long tail in a distribution of charge carrier arrival times (see, e.g., TAPD single-photon time resolution histogram at 905 nm with a slow component of 100 ns [83]). However, in a properly tuned detector configuration for fast timing applications this situation should be avoided.

Timing performance in terms of a response temporal profile is mostly defined by the detector and pixel capacitances, as discussed above. Obviously, the lower capacitances, the shorter response in all its components; sometimes this is a limiting and, therefore, game-changing factor.

In particular, for TOF applications, the short rise time of a single electron response (SER) is beneficial for single-photon time resolution because the steeper rising slope of the response translates electronic noise RMS into the lower timing jitter. And in the case of multiphoton pulse detection, for example, of scintillation flashes, the response is a convolution of the SER and the scintillation timing profile where the shorter the SER fall time, the steeper the rising slope of the multiphoton response [127,128].

#### 3.1.11. Radiation Hardness

Radiation hardness of the non-planar designs is rarely studied and is rather uncertain in general. Non-planarity does not prevent the detectors from radiation-induced degradation because the creation of point and cluster defects in the materials resulted in an excess dark generation and charge trapping as well as the so-called specific “acceptor removal effect” in silicon [88]. However, the sensitivity of the detector to degradation appeared to be different. A remarkable level of radiation hardness has been demonstrated by 3D silicon detectors (Section 2.9), and the first promising results have been observed for TAPD (Section 2.10).

### 3.2. Overall Performance in Detective Quantum Efficiency

**Figure 21:**
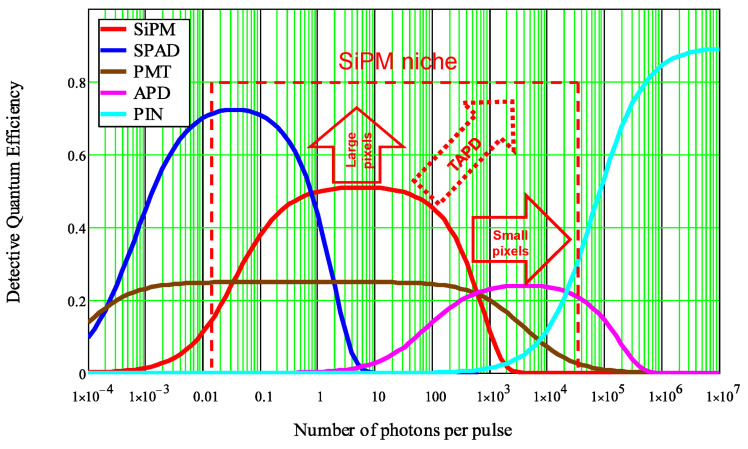
Detective quantum efficiency (DQE) vs. dynamic range in number of photons per pulse: a map of applicability and competitiveness of photodetectors. Reproduced with permission from [83]; published by Elsevier, 2023.

Detective quantum efficiency (DQE) is a well-known figure of merit of various detectors, which is defined as a squared ratio of the specific detector SNR to an ideal noiseless detector SNR defined by statistics of incident photons. In contrast to SNR, DQE represents internal properties of the detector excluding photon statistics. DQE depends on number of photons: it has a plateau inside a linear dynamic range, a decay at a low number of photons due to all kinds of noise, and a decay at a high number of photons due to non-linearity and saturation of the detector response. The plots of DQE vs. number of photons are very informative representations of the applicability and competitiveness of the detectors [30,31]. The DQE map for conventional photodetectors is shown in Figure 21, where the main trends in SiPM development are marked by solid arrows (large pixels for high DQE, and small pixels to obtain high dynamic range), and the new trend of non-planar designs represented by TAPD (high PDE and small pixels) is marked by dashed lines [83].

## 4. Conclusions

Starting from non-planar handcrafted semiconductor diodes in the 1930s, a planar design became the mainstream of the developments as the most perfect realization of the uniformity of all properties over the detector area. In parallel, non-planar designs have been applied to resolve some trade-off of the planar approach producing imperfect but original devices. Remarkable progress in semiconductor technologies, especially CMOS, allows us to make non-planar devices to be almost as perfect as the planar ones and, on the other side, squeezes dimensions of the planar devices to almost non-planar configurations.

Therefore, in the near future we can expect emerging developments of the non-planar designs in many directions and very promising synergy between planar and non-planar approaches.

## Data Availability

Not applicable.

## Abbreviations

The following abbreviations are used in this manuscript:

ADD	avalanche drift diode
APD	avalanche photodiode
BEOL	back end of line
BID	back illumination drift
BSI	backside-illumination
BW	bandwidth
CMOS	complimentary MOS
CPTA	Center of Perspective Technologies and Apparatus
CA-APD	current-assisted APD
CA-SPAD	current-assisted SPAD
CF-APD	charge-focusing APD
DAPD	discrete amplification APD
DQE	detective quantum efficiency
ENF	excess noise factor
FBK	Fondazione Bruno Kessler
FSI	frontside-illuminated
FWHM	full width at half maximum
LAAPD	large-area APD
MAPD	micropixel avalanche photodiode
MIS	metal-insulator-semiconductor
MOS	metal-oxide-silicon
MOSFET	MOS field effect transistor
MRS	metal-resistor-semiconductor
NAPD	nano-multiplication-region APD
NFAD	negative feedback APD
NIR	near-infrared
PDE	photon detection efficiency
PET	positron emission tomography
PDP	photon detection probability
RMS	root mean square
SACM	separate absorption, charge and multiplication
SER	single electron response
SDD	silicon drift detector
SiPM	silicon photomultiplier
SNR	signal-to-noise ratio
SPAD	single photon APD
SSPM	solid state photomultiplier
SWIR	shortwave infrared
TAPD	tip avalanche photodiode
TOF	time of flight
